# Medical and Physical Intelligence

**Published:** 1803-01-01

**Authors:** 


### MEDICAL AND PHYSICAL INTELLIGENCE.

### [FOREIGN AND DOMESTIC.]

---

PROGRAM *of the Prizes proposed by the* SOCIETY *of Montpellier, sitting in the School of Medicine.*

Subject of the first prize.—" To determine in what sorts and what circumstances of chronical maladies, inflammation may be useful or dangerous, and with what precautions we ought to excite or to moderate it, in the treatment of them?"

Attempts have been made to determine the utility and the danger of fever in chronical diseases. Inflammation exhibits a symptom, which has no small influence on certain maladies of this class. The Medical Society expects, that agreeably to a sufficient number of observations and decisive experiments, there should be established on this subject, clear, simple, comprehensive, and invariable principles, the application of which, it may be easy to reduce to practice. The relations of inflammation with chronical maladies ought to be considered under very different points of view. Sometimes, as an essential symptom of those maladies, it constitutes one of their principal elements, as appears in heavy and chronical inflammations of the viscera; then it is susceptible of error from excess or defect; and it often becomes necessary to lessen the inflammatory mode, or to reincite it *(relever.)* Sometimes, as a foreign symptom, it is accidentally developed during the course of the disease, as is observed in the affections of the lymphatic system, glandulous stoppages in the throat, cold, indolent, schirrous tumours, &c.; then it may become advantageous or hurtful, according to the time and the circumstances of its appearance.

It is therefore of importance to have fixed rules to excite or to moderate it in the treatment of it; and lastly, inflammation is sometimes the product of an acrid, heterogeneous, and virulent principle, fixed on a sensible part; and in this case, it is requisite to estimate its advantages or its inconveniences, its utility or its dangers, according to the knowledge that we have of the nature of that principle, on the contexture of the parts affected, on their importance and their sympathy with others, &c. This takes place in dartrous, venereal, scrophulous affections, where the inflammation assumes characters proper and relative to each. What are the real effects of inflammation in relation to those different orders of maladies? How can we distinguish whether it is useful or dangerous? What may be the favourable or prejudicial conditions in order to develope it? According to what views, with what pre-

 cautions, cautions, by what means, is it expedient to excite or to moderate it in these sorts of cases? The action of the atmospherical air, the impression of different gases, the injection of divers liquids, the application of the heat of the vescatory, caustics, cautery, *moxa*, the effectof compressive means, &c. All these things may be referred to the object of the question which the Society proposes; and ought, according to their respective degree of interest, to fix the attention of the candidates.

The prize will be to the value of 500 francs; it will be adjudged in the sitting of the 30th Floreal, year 11 of the Republic.

Subject of the second prize.— "To establish, agreeably to observation and experience, what is the degree of confidence that we ought to place in the method of administering, by frictions, different substances that are usually prescribed to be taken inwardly? In what relations are the effects produced by the same remedy taken inwardly, or applied in frictions? And what are the proportions that should be observed in the doses, to indicate the circumstances and the maladies which ought to give a preference to this method? And, lastly, in the different affections, what parts of the body should be fixed upon to apply this remedy with the most efficacy?"

The solution of this last question requiring, as it should seem, a series of observations and of experiments, which it would be difficult to collect or to make in the too short space of one year, the Society has conceived that it would be serving the candidates and science itself, by coming to a resolution that it would not adjudge the prize until its sitting of the 30th Floreal of the year 12. The value of it will be equal to that of the prize of the year 11. The resident Members of the Society are excluded from the competition for the two prizes.

The Memoirs, to be written in Latin or French, are to bear an epigraph or motto, which the author will take care to annex to the sealed billet which shall contain his name; they should come to hand before the 1st Floreal of the years in which the prizes will be adjudged, and should be directed, free of postage, to Cit. Lordat, the elder, perpetual Secretary of the Medical Society (Rue Blanquerie) of Bourdeaux.

The Society acknowledges, with gratitude, that it is indebted to the generosity of one of its Members, who wishes to remain unknown, for a sum of 200 francs, offered as a supplement to the prize of the year 11. As the question for the year 12 requires much investigation, the Society has thought that it will not be imposing upon the beneficence of the donor, to divide the said sum, so as to enlarge the two prizes equally.

---

A new analysis of the Boracites has been lately made by Cit. Vauquelin, the results of which are different from those of former chemists, chemists. This interesting mineral, which no where occurs but in the neighbourhood of Linebourg in the Electorate of Hanover, was first analysed by the celebrated German chemist, Mr. Westrumb of Hameln, who discovered in it:

| | |
|---|---|
| Boracic acid - - - | 68 |
| Magnesia - - - - | 13,05 |
| Lime - - - - - | 11 |
| Aluminous earth - - | 1 |
| Oxyd of iron - - - | 0,75 |
| Siliceous earth - - | 2 |
| | 95,80 |

In consequence of this analysis, it received the name of Borate Magneo-calcaire, by all the French chemists. Cit. Vauquelin, however, examining this substance some time ago with Mr. Smith, who had procured a considerable quantity of it, thought he had found, that lime was not an essential part in the composition of this mineral, as the powder of it made an effervescence with acids, which seemed to be exactly proportionable to the small quantity of lime that was present in this substance. In order to ascertain this circumstance, the two chemists endeavoured to separate the portion of carbonat from the borat by weak acids, diluted with much water, especially by the acetous acid; in which attempt, however, they could not properly succeed, as even the weakest acetous acid did likewise affect the Borat, on which account the question was left undecided for want of transparent crystals, which make no effervescence with acids. But some time after, Cit. Vauquelin being supplied with a sufficient quantity of perfectly transparent Boracits, through the medium of Dr. Stromeyer, of Gottingen, made new experiments with them, with the intention only of ascertaining the presence of lime. Having reduced them to powder, he added muriatic acid; in which, by means of a gentle heat, it was dissolved, and the whole mass afterwards evaporated to dryness, which removed the superabundant acid, and it was again dissolved in a small quantity of cold distilled water. By this method the greatest part of boracic acid was separated in form of white, brilliant laminae. Having diluted the above solution with more water, he mixed with it a certain quantity of oxalat of ammonia, which is known to be the best re-agent for discovering the presence of the smallest portion of lime that may be contained in any liquor, providing there is not a superabundance of acid in it. Notwithstanding this experiment, not the least traces of lime could be perceived in the said solution. For the purpose of being assured, whether the small quantity of boracic acid that was dissolved in the water, together with the muriat of magnesia, had not hindered the lime from being precipitated, he mixed a portion of muriat of lime, not exceeding $\frac{1}{30}$ of the Borat employed for the experiment, and instantly a cloud appeared throughout the liquor. On decomposing artificial Borat of lime in the same manner as the natural Borat, he obtained by the the addition of oxalat of ammonia a considerable precipitate. It appears therefore evident, that if the natural borat had contained only $\frac{1}{100}$ its weight of lime, some traces of it would have been discovered by the means which Cit. VAUQUELIN had employed; whence this chemist concludes, that the natural Borat Magnesien, which is perfectly transparent, contains no lime, but which however, in the opacous crystals, is admixed in form of carbonat, and is the cause of their opacity.

That mineral, therefore, should no longer be considered as a triple salt; and it ought not to have the appellation of Borat Magnesio-calcaire but only that of Borat Magnesien.

---

Dr. HUFELAND praises the excellent effects of the oil of the walnut kernel *(ol. nuc. Zugland)* in herpetic and other cutaneous complaints. He recommends it as one of the safest, most simple, and efficacious external remedies that can be resorted to, as it mitigates the pains and that burning sensation, sometimes almost insupportable, which accompany this obstinate disease; it never seems to have any ill effect if attention be given to the eruption suddenly disappearing, or during, as it is said, by the repulsion of the herpes, a circumstance which frequently happens by the application of metallic ointments, and which is often attended with much danger to the constitution; but although it cures the cutaneous affection in a short time, the cure is perfect and not followed by any bad consequences, providing the eruption does not originate in any obstinate internal or general disease. In a child, which was almost covered with a chronic and suppurating herpes, against which internal remedies, baths, and mercurial ointments had been employed without producing a perfect cure, the oil of walnut kernel was for some time used, and the eruption disappeared soon after. It is likewise an excellent remedy in small cutaneous eruptions, that are now and then observed in children. The oil ought to be fresh expressed, without heat, and applied to the affected places two or three times a day.

---

We find the Chenopodium Ambrosioides *(Botrys mexic. offic.)* very much recommended in HUFELAND's Journal in nervous diseases, spasms, paralyses, and asthmatic complaints. An old lady, who after an apopletic fit had retained a palsy of the tongue, to which the usual remedies had been applied without any benefit, was completely cured by this remedy; it proved likewise highly serviceable to a boy, who having had the small-pox, laboured under an obstinate nervous debility, and had lost the power of speech. The form in which it is to be used, is an infusion, or as a tincture.

---

The celebrated Dr. LENTIN, physician to his Britannic Majesty for Hanover, relates two cases of hydrops pectoris, in which the foxglove, digitalis purpurea, was administered with the greatest success.

cess. That obstinate disease, which frequently resists the most powerful remedies, seems to originate in an increased action of the vasa excernentia and in diminished action of the resorbent vessels. Digitalis seems of all others to be particularly adapted to the nature of that disease, as it diminishes the number of the pulse, and augments the oscillation of the resorbent vessels. The manner in which Dr. LENTIN uses this remedy is as follows: *Decoctum Digitalis Purpuerea.*

℞. Hb. digital. purpur. unc. j conc. coq. in aq. font. ℔ j. ß Colatur. unc. viij. admisce spirit. vin. rectific. unc. ß misc. et serv.

℞. Hujus decoct. unc. j. aq. menth. petrosel. aa. unc. ij. syrup. de althaea dr. ij. M. S. Every two hours a table spoonful to be taken.

---

The Professors of the Museum of Natural History in the garden of plants at Paris, have formed themselves into a Society, with a view of publishing whatever occurs new and interesting in this branch of national education. The extensiveness of the collection as it now stands, and the constant accumulation of new and interesting phenomena, will afford ample matter for their new publication, which they entitle, The Annals of the Museum of Natural History, the first Number of which has appeared, and which is to be repeated every Month; it consists of ten sheets and four plates, quarto size, so that this publication will form at the end of the year, two large quarto volumes of the newest matter in the several branches of Natural History; such as Comparative Anatomy, Chemistry, &c. The plates, which will be principally copied from Nature, and executed by Artists of known merit, cannot fail to become important to those who have not an extensive cabinet to resort to. In a future publication we mean to give a brief account of the contents of the first Number.

---

In a former Number (43) an anonymous Correspondent having asserted that Dr. Pearson had used Dr. Heberden's authority in his evidence at the House of Commons, for a statement which Dr. Heberden afterwards disallowed in giving his evidence, we are authorized to print the following explanation, communicated by Dr. Heberden to Dr. Pearson.

"Dr. Heberden acquiesces in the correctness of his printed evidence, with the addition of only two words, viz. "having heard nothing of it *from him*." In fact, Dr. H. was acquainted with Mr. Battiscombe, but received his information respecting the vaccine inoculation from another quarter. Still it is true, that when Dr. Heberden mentioned the circumstance to Dr. Pearson, upon Dr. Lind's authority, he corroborated his statement, by adding that Dr. Gisborne had been made acquainted with the same account through Mr. Battiscombe. So that Dr. H. may, in effect, be said to have related the circumstance to Dr. Pearson upon the united testimony of Dr. Lind and Mr. Battiscombe; though his information in the latter case not having been derived *immediately* from that gentleman, gentleman, he could not, with propriety, produce to the Committee the authority of Mr. Battiscombe for what he had heard upon the subject."

---

Dr. BATTY's Course of Lectures on the Theory and Practice of Midwifery, will commence on Monday, January 17, 1803, at eleven o'clock in the forenoon, at his house in Great Marlborough Street.

---

Mr. MACARTNEY will commence his Lectures upon Comparative Anatomy and Physiology, at St. Bartholomew's Hospital, on Monday, the last day of this month, at eight o'clock in the evening: he intends also to include the structure of Vegetables, as being illustrative of the laws of organic existence. For further particulars apply to Mr. Nicholson, at the Apothecary's Shop, St. Bartholomew's Hospital.

---

Mr. HEADINGTON and Mr. FRAMPTON will commence their Second Course of Lectures on Anatomy, Physiology and the Principles and Operations of Surgery, on Thursday the 20th of January, 1803, at 9 o'clock in the morning.

Private teaching under the direction of Mr. Armiger.

During the Winter, a Course of Lectures on Surgery, illustrated by cases received into the Hospital, will be delivered by Mr. Blizard, Mr. J. Blizard, and Mr. Headington.

---

Mr. CHEVALIER, Surgeon to the Westminster General Dispensary, will begin his next Course of Lectures on the Principles and Operations of Surgery, on Monday the 31st of January, at 7 o'clock in the evening, at his house in South Audley-street, Grosvenor-square, where printed particulars may be had.

---

Messrs. WAGSTAFFE and SYER will commence their Spring Course of Lectures on the Elements and Practice of Midwifery, on Monday tho 3d of January, 1803, at a quarter before seven in the evening, at their Lecture-room, No. 102, Leadenhall-street.

---

A THIRD VOLUME OF MEDICINA NAUTICA, will be published in the course of a few days. Some new and curious particulars in the History of Health, are said to form a part of this interesting Volume.

---

Dr. WALKER, Physician to the City of London Lying-in Hospital, has published General Observations on the Constitution of Women, and on some of the Diseases to which they are more particularly liable.

---

[ W. Thorne, Printer, Red Lion Court, Fleet Street. ]

